# Impact of withholding early parenteral nutrition in adult critically ill patients on ketogenesis in relation to outcome

**DOI:** 10.1186/s13054-021-03519-3

**Published:** 2021-03-11

**Authors:** Astrid De Bruyn, Lies Langouche, Sarah Vander Perre, Jan Gunst, Greet Van den Berghe

**Affiliations:** Clinical Division and Laboratory of Intensive Care Medicine, Department of Cellular and Molecular Medicine, KU Leuven, 3000 Leuven, Belgium

Withholding parenteral nutrition until one week after intensive care unit (ICU) admission (late-PN) was previously shown to accelerate recovery and reduce infections in critically ill adults and children, as compared to early supplementing insufficient enteral feeding with parenteral nutrition (early-PN) [1, 2]. In a detailed secondary analysis of the pediatric PEPaNIC randomized controlled trial (RCT), we previously identified enhanced ketogenesis as potential mediator of part of this outcome benefit. Indeed, late-PN increased plasma concentrations of the ketone 3-hydroxybutyrate (3HB) up to sixfold, with a peak effect on day 2 [3]. Increased 3HB independently associated with an accelerated weaning from mechanical ventilation and a shorter time to live ICU discharge. These associations remained significant after adjusting for ketogenic regulators, suggesting a direct mediator role [3].

In this secondary analysis of patients included in the EPaNIC RCT (*n* = 4640), we studied whether late-PN had a similar impact on ketones in critically ill adults as compared with early-PN, and whether this may have mediated its outcome benefits. To this purpose, we quantified plasma 3HB with an enzymatic assay [3] in the predefined subgroup of patients with a surgical contraindication to enteral nutrition [1]. In this subgroup (509/517 patients with available plasma), there was a larger difference in caloric intake than in the total study population (Fig. 1a), and the outcome benefits of late-PN appeared to be more pronounced [1] (Table1).

**Fig. 1:**
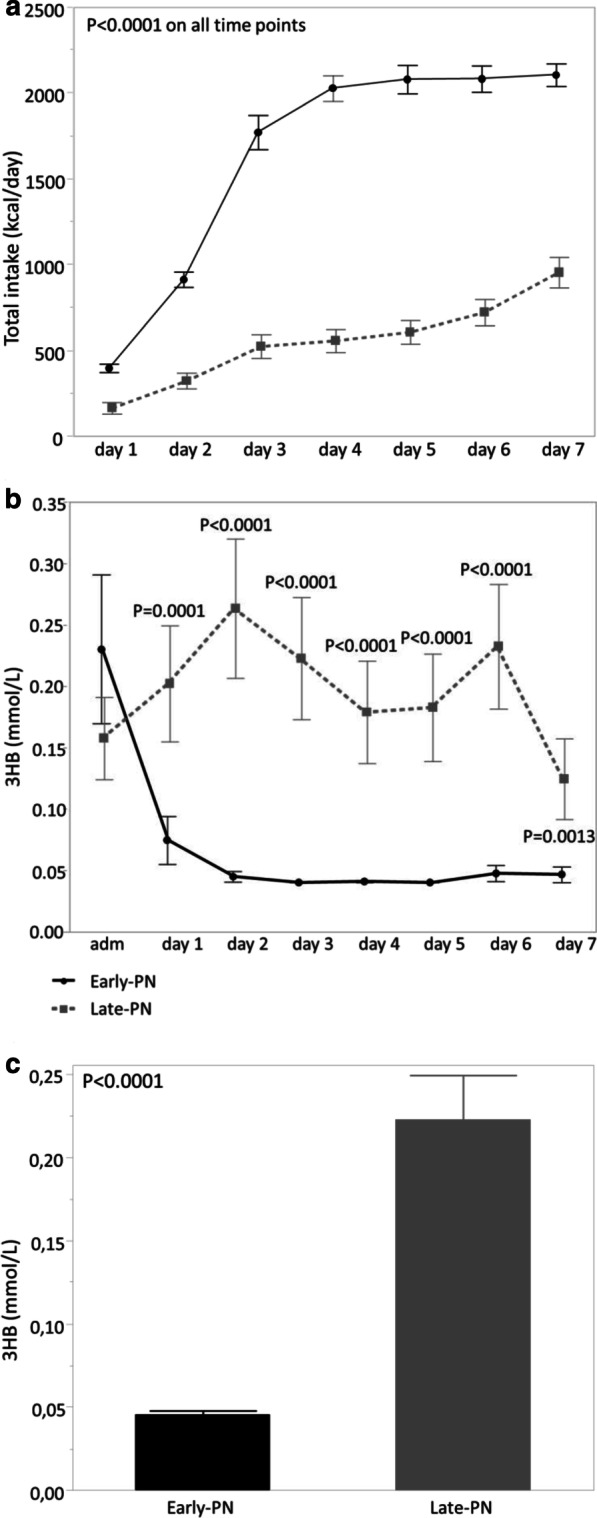
Total caloric intake (**a**) and plasma 3HB (**b**) from admission/day 1 until day 7 in a matched subset of early-PN patients (*n* = 55) and late-PN patients (*n* = 55) with a surgical contraindication to enteral nutrition, an ICU stay of at least 7 days, and available plasma sample on each day. The groups were propensity score-matched for age, BMI, malignancy, APACHEII score, NRS score, diagnostic group. **c** Plasma 3HB concentration on day 2 in ICU (or day 1 for 65 patients with a shorter ICU stay) in the total cohort of patients with surgical contraindication to enteral nutrition (*n* = 509). Plasma 3HB concentrations were significantly higher (*P* < 0.0001) in late-PN than in early-PN patients. Data are shown as means ± standard errors

**Table 1:** Baseline characteristics and outcome of the study patients

EPaNIC patients with surgical contraindication to enteral nutrition and available plasma	Early-PN	Late-PN	P-value
Baseline characteristics time cohort (n = 110)	*n* = 55	*n* = 55	
Age (years)—median (IQR)	68.1 (55.9–77.8)	66.1 (55.5–73.6)	0.45
BMI—median (IQR)^a^	25.3 (22.2–29.2)	25.7 (22.4–33.7)	0.61
Malignancy—no. (%)	31 (56.3)	32 (58.1)	1.00
APACHEII score—median (IQR)^b^	36 (28–39)	35 (31–39)	0.75
NRS score—median (IQR)^c^	4 (3–6)	4 (4–6)	0.49
Diagnostic categories			0.69
(Complications after) abdominal/pelvic surgery—no. (%)	36 (65.4)	33 (60.0)	
(Complications after) thoracic surgery—no. (%)	19 (34.5)	22 (40.0)	
Baseline characteristics total cohort (n = 509)	*n* = 252	*n* = 257	
Age (years)—median (IQR)	64.4 (54.4–73.5)	64.4 (53.7–73.0)	0.99
BMI—median (IQR)^a^	24.5 (22.2–28.3)	24.6 (22.0–28.7)	0.89
Malignancy—no. (%)	161 (63.8)	154 (59.9)	0.36
APACHEII score—median (IQR)^b^	27 (16–37)	28 (18–36)	0.86
NRS score—median (IQR)^c^	4 (3–6)	4 (3–5)	0.34
Diagnostic categories			0.70
(Complications after) abdominal/pelvic surgery—no. (%)	168 (66.6)	177 (68.8)	
(Complications after) thoracic surgery—no. (%)	84 (33.3)	80 (31.1)	
Outcome of total cohort (n = 509)	*n* = 252	*n* = 257	
Hazard ratio (95% CI) for time to live ICU discharge		1.23 (1.02–1.48)	0.024
Alive ICU discharge within 8 days—no. (%)	125 (49.6)	151 (58.7)	0.041
New infection—no. (%)	103 (40.2)	78 (29.8)	0.009

Data are presented as frequencies and percentages or medians with interquartile ranges. Fisher’s exact test and Kruskal–Wallis test were used to analyze univariable differences between patient groups, as appropriate. Hazard ratio and 95% confidence interval (CI) was calculated with the use of Cox proportional-hazard analysis of the effect of late-PN, with adjustment for age, BMI, malignancy, APACHEII score, NRS score, and diagnostic category

^a^The body-mass index is the weight in kilograms divided by the square of the height in meters

^b^Scores on the Acute Physiology and Chronic Health Evaluation II (APACHE II) range from 0 to 71, with higher scores indicating a greater severity of illness

^c^Scores on Nutritional Risk Screening (NRS) range from 0 to 7, with higher scores indicating a higher risk of malnutrition

We first performed a time course analysis in a propensity-score-matched subset of 110 patients (Table 1), to study whether late-PN enhanced ketogenesis and to identify the day of maximal effect (if any) (Fig. 1b). In the matched subset, late-PN significantly increased plasma 3HB from day 1 until day 7 (all *P* ≤ 0.0013), with a maximal effect on day 2. Subsequently, we quantified plasma 3HB on this day of maximal effect in all patients with a surgical contraindication to enteral nutrition. In these patients, late-PN significantly (*P* < 0.0001) increased plasma 3HB. Thereafter, we studied a potential mediator role of this 3HB effect on time to live ICU discharge, live ICU discharge within 8 days, and incidence of new infection through multivariable Cox, respectively logistic regression analysis, adjusted for baseline risk factors (age, BMI, malignancy, APACHEII score, NRS score, diagnostic group). Plasma 3HB did not independently associate with time to live ICU discharge (*P* = 0.54), live ICU discharge within 8 days (*P* = 0.23) or incidence of new infection (*P* = 0.71).

We demonstrated that withholding early parenteral nutrition induced ketogenesis in adult ICU patients with a surgical contraindication to enteral nutrition [3]. However, ketone concentrations were only modestly elevated as compared to the much larger effect in critically ill children, and in contrast to critically ill children, plasma 3HB did not independently associate with enhanced recovery. Also in health, the ketogenic response is known to be more pronounced in children than in adults [4]. Although speculative, this may explain why critically ill children had a more pronounced outcome benefit than adults [1, 2]. Also in critically ill children, however, there was no independent association of 3HB with incidence of infections, which suggests that other mechanisms are involved in outcome protection through late-PN. In this regard, we previously identified increased autophagy as one potential mediator [5]. Of note, especially in early-PN adult patients, a considerable number of 3HB measurements were assigned the detection limit (0.04 mmol/L) due to lower concentrations, which may have obscured detecting a mediating role of ketones on outcome.

In conclusion, withholding early parenteral nutrition enhanced ketogenesis in critically ill adults, but in contrast to children, increased ketones did not explain the improved outcome. This suggests clinical benefits of omitting early-PN were mediated through other mechanisms.

## Data Availability

Data sharing is offered under the format of collaborative projects. Proposals can be directed to the corresponding author.
